# The Crosstalk between the Gut Microbiota and Mitochondria during Exercise

**DOI:** 10.3389/fphys.2017.00319

**Published:** 2017-05-19

**Authors:** Allison Clark, Núria Mach

**Affiliations:** ^1^Health Science Department, Open University of CataloniaBarcelona, Spain; ^2^UMR 1313, INRA, AgroParisTech, Université Paris-SaclayJouy-en-Josas, France

**Keywords:** gut microbiota, energy, endurance, inflammation, mitochondria, oxidative stress

## Abstract

Many physiological changes occur in response to endurance exercise in order to adapt to the increasing energy needs, mitochondria biogenesis, increased reactive oxygen species (ROS) production and acute inflammatory responses. Mitochondria are organelles within each cell that are crucial for ATP production and are also a major producer of ROS and reactive nitrogen species during intense exercise. Recent evidence shows there is a bidirectional interaction between mitochondria and microbiota. The gut microbiota have been shown to regulate key transcriptional co-activators, transcription factors and enzymes involved in mitochondrial biogenesis such as *PGC-1*α*, SIRT1*, and *AMPK* genes. Furthermore, the gut microbiota and its metabolites, such as short chain fatty acids and secondary bile acids, also contribute to host energy production, ROS modulation and inflammation in the gut by attenuating TNFα- mediated immune responses and inflammasomes such as *NLRP3*. On the other hand, mitochondria, particularly mitochondrial ROS production, have a crucial role in regulating the gut microbiota via modulating intestinal barrier function and mucosal immune responses. Recently, it has also been shown that genetic variants within the mitochondrial genome, could affect mitochondrial function and therefore the intestinal microbiota composition and activity. Diet is also known to dramatically modulate the composition of the gut microbiota. Therefore, studies targeting the gut microbiota can be useful for managing mitochondrial related ROS production, pro-inflammatory signals and metabolic limits in endurance athletes.

## Introduction

Endurance exercise can be defined as cardiovascular exercise—such as running, cross-country skiing, cycling, aerobic exercise or swimming—that is performed for an extended period of time (Joyner and Coyle, 2008; Mach and Fuster-Botella, in press). Endurance exercise requires a great amount of physiological adaptations in order to keep up with energy demands and to maintain the body's homeostasis (Mach and Fuster-Botella, in press). The main physiological changes that occur during intense exercise include: (i) coordinated muscle contractions (Spriet and Watt, 2003), (ii) glucose and fatty acid oxidation (Spriet and Watt, 2003), (iii) increased use of glucagon stores (Spriet and Watt, 2003), (iv) oxidative phosphorylation (Befroy et al., 2008), (v) mitochondrial biogenesis in different tissues, including muscles (Radak et al., 2008), (vi) electrolyte and temperature rebalance, (vii) increased production of reactive oxygen species (ROS) and reactive oxygen nitrogen species (RONS), vii) activation of the sympatho-adreno-medullary and hypothalamus-pituitary-adrenal (HPA) axes, which results in the release of stress hormones into these circulatory system (reviewed by Clark and Mach, 2016) as well as systemic inflammation and immune responses (Mach and Fuster-Botella, in press). In some cases, gastrointestinal hypoxia and hypoperfusion increase intestinal permeability and oxidative stress in the gastrointestinal tract (Magalhães et al., 2013; Clark and Mach, 2016).

The role mitochondria play during endurance exercise has been expanded beyond the scope of its energy producing capacity. Each mammalian cell contains hundreds to thousands of mitochondria, and the organelle's size, shape, and number depend on various physiological conditions and stimulus such as endurance exercise, high temperature, diet or hormones (Bartlett and Eaton, 2004; Knuiman et al., 2015; Busquets-Cortés et al., 2016). Mitochondria are organelles that are the primary energy centers, oxidizing fats and sugars to generate adenosine triphosphate (ATP). Mitochondrial oxidative phosphorylation (OXPHOS), which combines electron transport with cell respiration and ATP synthesis (Lee and Wei, 2005; Cheng and Ristow, 2013) and fatty acid β-oxidation (FAO) are the two metabolic pathways that are central to this process. Mitochondria can also use enzymatic pathways of the tricarboxylic acid (TCA) cycle to generate ATP (about 20% of ATP; Papa et al., 2012). These organelles are also involved in other essential metabolic and cellular processes, including calcium homeostasis, intracellular signaling, heme biosynthesis, and acute cell death (Wai and Langer, 2016). As a side product of normal respiration, mitochondria produce reactive ROS and RONS (Green et al., 2011), which have important roles in cell signaling and homeostasis, but excessive amount of ROS could also cause significant damage to cell structures and induce cytokines release or cell death by apoptosis (Green et al., 2011). Moreover, as they replicate, their genomes accumulate mutations that eventually compromise the efficiency of OXPHOS (Green et al., 2011). Mitochondria also play a central role in the initiation of inflammation through inflammasomes, a molecular set of functions that activate caspase-1, which facilitates the secretion of the inflammatory cytokines IL-1, IL-18, and other inflammatory mediators (Green et al., 2011).

It is clear that mitochondrial functions are important during high metabolic activities such as endurance exercise. Compared to other athletes, endurance athletes have a higher number and volume of mitochondria in the skeletal muscle in order to meet energy needs (Befroy et al., 2008; Hood et al., 2011; Busquets-Cortés et al., 2016). An increase in biogenesis has been shown to improve muscle endurance performance due to its increased capacity for OXPHOS and β-oxidation of fatty acids or ketone bodies and thus energy production (Hood et al., 2011).

In endurance athletes, moderate ROS and RONS production has been shown to stimulate mitochondrial biogenesis and FAO (Wai and Langer, 2016). However, redox imbalance during prolonged periods of time has been associated with a rapid onset of fatigue, the inability to maintain the speed and intensity of performance (Rapoport, 2010). Additionally, strenuous exercise causes an increase in the number of pro-inflammatory cytokines, such as TNFα, IL-1, IL-6, anti-inflammatory modulators and macrophage inflammatory protein-1, indicating a dose-response effect between biological responses to exercise and host immunity (reviewed by Mach and Fuster-Botella, in press). Due to the key role of mitochondria have in the activation of inflammasomes and other inflammatory responses, special attention is given to mitochondria during endurance exercise.

New research shows a bidirectional communication exists between the gut microbiota and mitochondria (Ma J. et al., 2014; Mottawea et al., 2016; Saint-Georges-Chaumet and Edeas, 2016). The gut microbiota contains more than 100 trillion microorganisms (Rajilić-Stojanović and de Vos, 2014), which comprise approximately 160 species and 9 million genes (Li et al., 2014). The gut microbiota are key to host metabolism as they aid in the digestion and absorption of food (Neis et al., 2015), neutralize drugs and carcinogens, synthesize choline (Nicholson et al., 2012), secondary bile acids (Hylemon et al., 2009; Sagar et al., 2015; Joyce and Gahn, 2016), folate (Sugahara et al., 2015), vitamin K2 (Marley et al., 1986) and short chain fatty acids (SCFA). Additionally, the gut microbiota protects the host against pathogenic infection (Lozupone et al., 2012), stimulates and matures the immune system (Vighi et al., 2008) and epithelial cells (Hooper and Gordon, 2001) and regulates oxidative stress (Xu et al., 2014).

The interaction between microbiota and mitochondria appears to occur primarily through signaling from the gut microbiota to mitochondria and from mitochondria to the gut microbiota by means of endocrine, immune, and humoral links (Mottawea et al., 2016). The most direct evidence of mitochondrial-microbiota interactions have come from the studies about mitochondrial functions that are affected during bacterial infection as well as different strategies developed by bacterial pathogens to subvert functions related to calcium homeostasis, maintenance of redox status and mitochondrial morphology (reviewed by Lobet et al., 2015). Pathobionts (i.e., *Fusobacterium, Veillonella*, and *Atopobium parvulum*) tend to control mitochondrial activity in favor of infection and inflammation through the production of hydrogen sulfide (H_2_S) and nitrogen oxide (NO) (Mottawea et al., 2016). Some other recent studies demonstrated that metabolites produced by commensal gut microbiota, including the beneficial SCFA and secondary bile acids, might influence mitochondrial functions related to energy production, mitochondrial biogenesis, redox balance and inflammatory cascades, making it a potential therapeutic target for endurance (Circu and Aw, 2012; Bär et al., 2013; den Besten et al., 2013; Mottawea et al., 2016). For instance, gut commensal microbiota reduce ROS production via SCFA such as N-butyrate (Mottawea et al., 2016).

On the other hand, mitochondrial functions might modify the gut microbiota composition and activity because they are able to induce innate immune responses (Green et al., 2011) when infectious microorganisms and cellular damage are detected. Mitochondria also influence the activities of intestinal functional effector cells, such as immune cells, epithelial cells and enterochromaffin cells (Cunningham et al., 2016). These same cells, on the other hand, are under the influence of the gut microbiota, whose contributing role in mitochondria functions is becoming evident. Lastly, polymorphisms of mitochondrial genes such as *ND5*, and *CYTB* genes or D-Loop region in the mitochondrial genome have been associated with specific gut microbiota compositions (Ma J. et al., 2014).

Due to the high physiological demands and adaptations needed during intense exercise as well as the growing importance the gut microbiota-mitochondria crosstalk has for the host's overall intestinal health, energy production, immune response, mitochondrial biogenesis, and redox balance, this review will focus on the available evidence supporting the existence of interaction between mitochondria and microbiota, as well as the possible physiological mechanisms involved during endurance exercise.

## Materials and methods

We conducted a systematic review and synthesis of relevant qualitative research according to the requirements established in the preferred reporting items for systematic review and meta-analysis protocols (Shamseer et al., 2015). The protocol was registered a priori with PROSPERO on February 8, 2017 with the ID number CRD42017056852.

### Eligibility criteria and literature search strategy

A systematic and comprehensive search of electronic databases, including MEDLINE, Scopus, ClinicalTrials.gov, the PROSPERO International Prospective Register of Systematic Reviews, Science Direct, Springer Link, and EMBASE was done from January 2017 to April 2017.

The following keywords were used in our search: “mitochondria,” “mitochondrial biogenesis,” “oxidative phosphorylation,” “oxidative stress,” “gut microbiota,” “short chain fatty acids,” “microbiota metabolites,” “endurance exercise,” “inflammation,” “PCG-1α,” “AMPK,” and “SIRT1.” The search was not restricted to the type of study (i.e., species, meta-analysis, case-control, prospective cohort studies, reviews), sample size, year of publication, publication status or follow-up; however, we only consulted articles published in English and did not include any doctorate thesis. Bibliographies of the identified reviews and original research publications were hand-selected for additional studies that may have been missed by the database searches. All articles were exported to the reference database Zotero. Due to the nature of this review, no request was performed for the ethics committee's approval.

### Data extraction and synthesis

Full copies of citations coded as potentially relevant were obtained, and those meeting the inclusion criteria were read in detail and data extracted. One reviewer (AC) extracted information about the study aim, population and sample size, experimental design, and duration of follow-up, specie, individual characteristics, and changes in the gut microbiota composition, energy metabolism, redox activity and immune response and association or not with mitochondrial function during endurance exercise. The primary outcome was the crosstalk occurred between the gut microbiota and mitochondria in response to intense exercise that includes: the gut microbiota's regulation of key mediators in mitochondrial biogenesis (i.e., AMPK, PCG-1α, SIRT1) as well as exercise-induced oxidative stress and inflammation in the gut. Details were then checked by a second reviewer (NM). If eligibility could be determined, the full article was retrieved.

The articles and extracted data were read and the findings organized by: (i) mitochondrial functions involved in energy production, ROS production and inflammation during endurance exercise; (ii) experimental studies about the possible crosstalk between mitochondrial function and the gut microbiota; (iii) experimental studies that demonstrated a possible link between mitochondrial functions and changes in the gut microbiota profiling in response to endurance exercise; (iv) experimental studies or reviews that showed a relationship between the roles the genetic variants in mitochondrion genome play in gut functions and microbiota profiling.

### Data synthesis

A search conducted in January 2017 resulted in the following list of key terms combinations (gut microbiota and energy production = 19; microbiota and oxidative stress = 22; mitochondria and oxidative stress = 16; mitochondria, microbiota, endurance exercise = 1). A total of 77 experimental studies and 84 reviews met the inclusion criteria and were included in the review. Most of the articles were reviews or randomized controlled trials. Periods of data collection spanned from 1980 to 2017, proving data from humans and animals models (i.e., mice, rats, horses, cats).

## Discussion

### The bidirectional crosstalk between the gut microbiota and mitochondrial functions

Mitochondria are dynamic organelles whose quantity and volume changes in response to cellular oxidative and metabolic demands. Although the mitochondrial genome is small, mitochondrial DNA encodes genes that might be essential for energy production, redox balance and inflammation regulation during endurance exercise. The human mitochondrial genome is a 16.6 kb circular DNA that encodes 13 peptides involved in OXPHOS, two ribosomal, and 22 transfer RNA that are crucial for intra-mitochondrial protein synthesis. Mitochondria functions are under dual genetic control of both the mitochondrial genome and the nuclear genome. It is known that more than 1,500 genes encoded by nuclear genome (Stewart and Chinnery, 2015) intervene in the mitochondrial functions through a complex orchestration of transcriptional and translational mechanisms of genes and non-coding RNAs (Shock et al., 2011).

During endurance exercise, the co-activator such as *PGC-1*α and transcription factors nuclear respiratory factor 1 and 2 (*NRF1, NFR2*) (Wu et al., 1999; Hood et al., 2011), thyroid hormone tri-iodothyronine (T3) receptor p43, cyclic-AMP response element binding protein (*CREB*), tumor suppressor p53, signal transducer and activator of transcription 3 (*STAT3*) and the estrogen receptors all control mitochondrial function (Hood et al., 2011). Among them, *PGC-1*α has been reported to be the most dominant regulator of mitochondrial function and respiration in muscles (Hood et al., 2011), especially during endurance exercise (Steinberg et al., 2006; Wright et al., 2007; Lira et al., 2010). During exercise, *PGC-1*α has been found to be up regulated and increase mitochondrial electron transport chain but also the mitochondrial DNA copy numbers through the activation of cyclooxygenase (COX) subunit II and COX unit IV (Safdar et al., 2011). Additionally, *PGC-1*α is involved in thermogenesis, glucose metabolism and oxidative capacity in various tissues and can be phosphorylated by 5′ adenosine monophosphate-activated protein kinase (AMPK), an enzyme also involved in mitochondrial biogenesis. AMPK is activated by cytokines and exercise primarily in response to changes in the AMP: ATP ratio (Lim et al., 2010) and activates *NRF1* and *NRF2* (Lee and Wei, 2005). Moreover, silent regulator 1 (*SIRT1*), a redox sensitive energy sensor, can also affect mitochondrial biogenesis via *PGC-1*α deacetylation (Lakhan and Kirchgessner, 2010; Radak et al., 2013), as well as muscular-fiber switching (Huang et al., 2016).

Beyond the nuclear and mitochondrial genome regulation of mitochondria functions, the genetic information encoded in all the microorganisms acquired from the environment (collectively known as the microbiome) also regulate mitochondrial functions by modifying energy production, ROS production, inflammatory responses and transcription factors involved in mitochondrial biogenesis. By definition, individual strains of a bacterial species can differ by up to 30% in terms of genetic sequence (Zhao, 2010). Considering that the genomes of humans and mice differ by only 10%, the genetic and functional diversity within the same bacterial species can be overwhelmingly high (Zhao, 2010). Moreover, the contribution made by these microorganisms becomes truly impressive considering only 10% of the total number of cells in human body consists of human cells, with the rest coming from symbiotic bacterial cells (Zhao, 2010).

Phylogenic analyses based on genes located in the mitochondrial genome indicate that mitochondria are of bacterial origin having evolved from α–proteobacteria (Gray et al., 2001). Various parasites from genera such as *Rickettsia* (Andersson et al., 1998), *Ehrlichia* and *Anaplasma* are believed to be the closes eubacterial relatives of mitochondria (Gray et al., 1999). Although most of the genes of ancestral α–proteobacteria have disappeared from the mitochondrial genome, there appears to be a close monophyletic lineage between cytochromes employed by bacteria and mitochondria such as cytochrome oxidases illustrating that the aerobic respiratory chain could be bacterial in origin (Kurland and Andersson, 2000). Other components of the mitochondrial proteome that are derived from α–proteobacteria that have been transferred to nuclei are ATP synthase 1 and 3 (*atp*1 and *atp*3) (Kurland and Andersson, 2000). As such, mitochondria have a separate genome and provide the oxygen consumption–driven synthesis of adenosine triphosphate (ATP) (via oxidative phosphorylation, OXPHOS).

**Figure 1:**
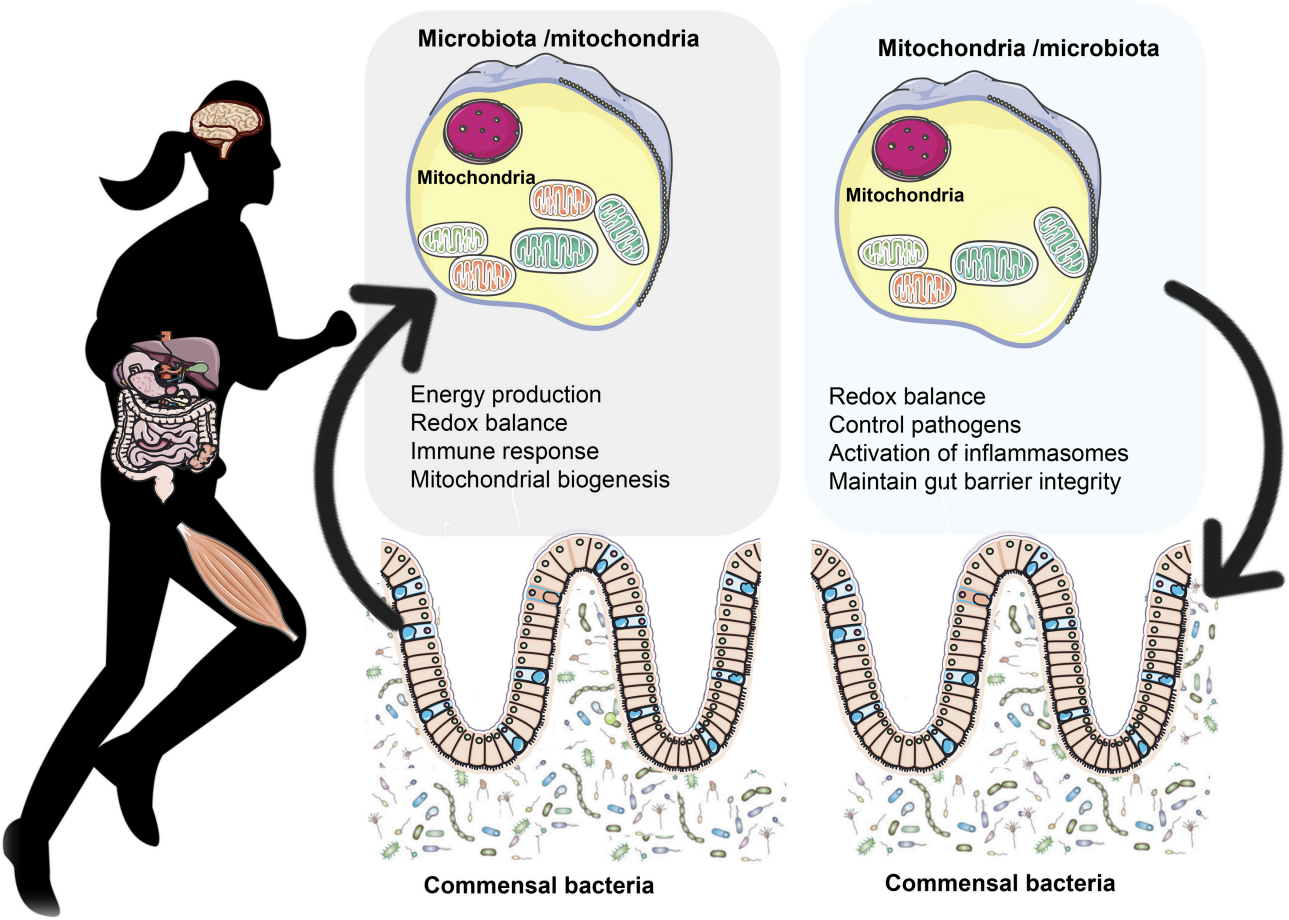
**The bidirectional crosstalk between the gut microbiota and mitochondria**. Gut microbiota to mitochondria crosstalk: Recent evidence shows there is a bidirectional crosstalk between the gut microbiota and mitochondria. Microbiota and their byproducts (SCFA and secondary bile acids) regulate redox balance and energy production. Secondary bile acid metabolism might also directly modify mitochondrial biogenesis, inflammation and intestinal barrier function in different types of cells (Gao et al., 2009; Korecka et al., 2013; Alex et al., 2014; Caron et al., 2014; Kazgan et al., 2014). In the mitchondria of colonocytes, butyrate undergoes FAO which produces acetyl-CoA that enters the TCA cycle resulting in ATP and CO_2_ (Donohoe et al., 2011). Among the SCFA, butyrate is a key regulator of energy production and mitochondrial function by inducing PGC-1α gene expression in skeletal muscles and brown adipose tissue (Gao et al., 2009) and improving respiratory capacity and FAO via AMPK-ACC pathway activation (Mollica et al., 2017). **Mitochondria to microbiota crosstalk:** Mitochondria regulate gut functions (Igarashi and Guarente, 2016; Wang et al., 2016), such as intestinal barrier protection (Peng et al., 2009) and mucosal immune response, which help maintain the mucus layer (Ma Y. et al., 2014) and intestinal microbiota (Shimada et al., 2012; Caron et al., 2014). *SIRT1* maintains intestinal barrier function through various mechanisms such as enhancing crypt proliferation and suppressing villous apoptosis (Wang et al., 2012), stimulating intestinal stem cell expansion in the gut (Igarashi and Guarente, 2016), regulating tight junction expression of zonulin ocludin-1, occludin and claudin-1 during hypoxia (Ma Y. et al., 2014). Mitochondrial genome variants may affect the gut microbiota composition. For example, polymorphisms in the *ND5*, and *CYTB* genes or D- Loop region of mitochondrial genome have been associated with specific gut microbiota compositions like *Eubacterium* and *Roseburia*, which are butyrate producers (Ma Y. et al., 2014). Additionally, the European haplotype HV has been associated with decreased odds of severe sepsis, higher OXPHOS capacity and ROS and RONS production (Jiménez-Sousa et al., 2015) as well as elevated VO_2max_ and aerobic ATP production in response to exercise (Martinez et al., 2010).

Lobet et al. (2015) suggested that mitochondria are a target of choice for bacterial pathogens as they are not only a key component of the central metabolism but they also take part to cell signaling through ROS production and control of calcium homeostasis as well as cell apoptosis. However, beyond bacterial infection, in the last years there have been some experiments conducted mainly on animals aimed to explore how the commensal gut microbiota modulates mitochondrial functions (Ma Y. et al., 2014; Mottawea et al., 2016; Saint-Georges-Chaumet and Edeas, 2016). Overall, these studies have shown that mitochondria respond to the commensal gut microbiota through three main ways: (i) regulating energy production, (ii) altering redox balance, and (iii) regulating immune reactions by attenuating TNFα-induced and inflammation-induced oxidation that lead to mitochondrial dysfunction. Given the widespread belief that mitochondria are symbionts of ancient α–proteobacteria origin, the interrelationship between mitochondrial functions and microbiota is of great interest (Figure 1).

On the other hand, mitochondria might modify the commensal microbiota composition and pathogen colonization and adherence through various mechanisms: (i) production of ROS and RONS, (ii) induction of the secretion of immune cells and enterochromaffin cells, (iii) modulation of gut functions, such as intestinal barrier function and appropriate mucosal immune response, all important for the maintenance of the mucus layer and biofilm where individual groups of bacteria grow, (iv) mitochondrial genetic variants and heteroplasmy (Figure 1).

### How the gut microbiota modulates mitochondrial functions

#### The gut microbiota's regulation of mitochondrial energy production

A primary adaptation endurance athletes possess compared to the nonathletic population is mitochondria biogenesis and improved VO_2_ max, which enables better oxygen uptake, OXPHOS and FAO in skeletal muscles (Rivera-Brown and Frontera, 2012). Endurance exercise is the most potent physiological inducers of mitochondrial biogenesis. Regular endurance training within 4–6 weeks in humans and mammals has been shown to increase mitochondrial content from 30 to 100% (Hood et al., 2011) and to increase the volume density up to 40% (Hood, 2001; Lundby and Jacobs, 2016). In the same line, 5 months of endurance exercise induced systemic mitochondrial biogenesis, prevented mitochondrial DNA depletion and mutations, increased mitochondrial oxidative capacity and respiratory chain assembly, restored mitochondrial morphology, and blunted pathological levels of apoptosis in multiple tissues of mitochondrial DNA mutator mice (Safdar et al., 2011).

**Figure 2:**
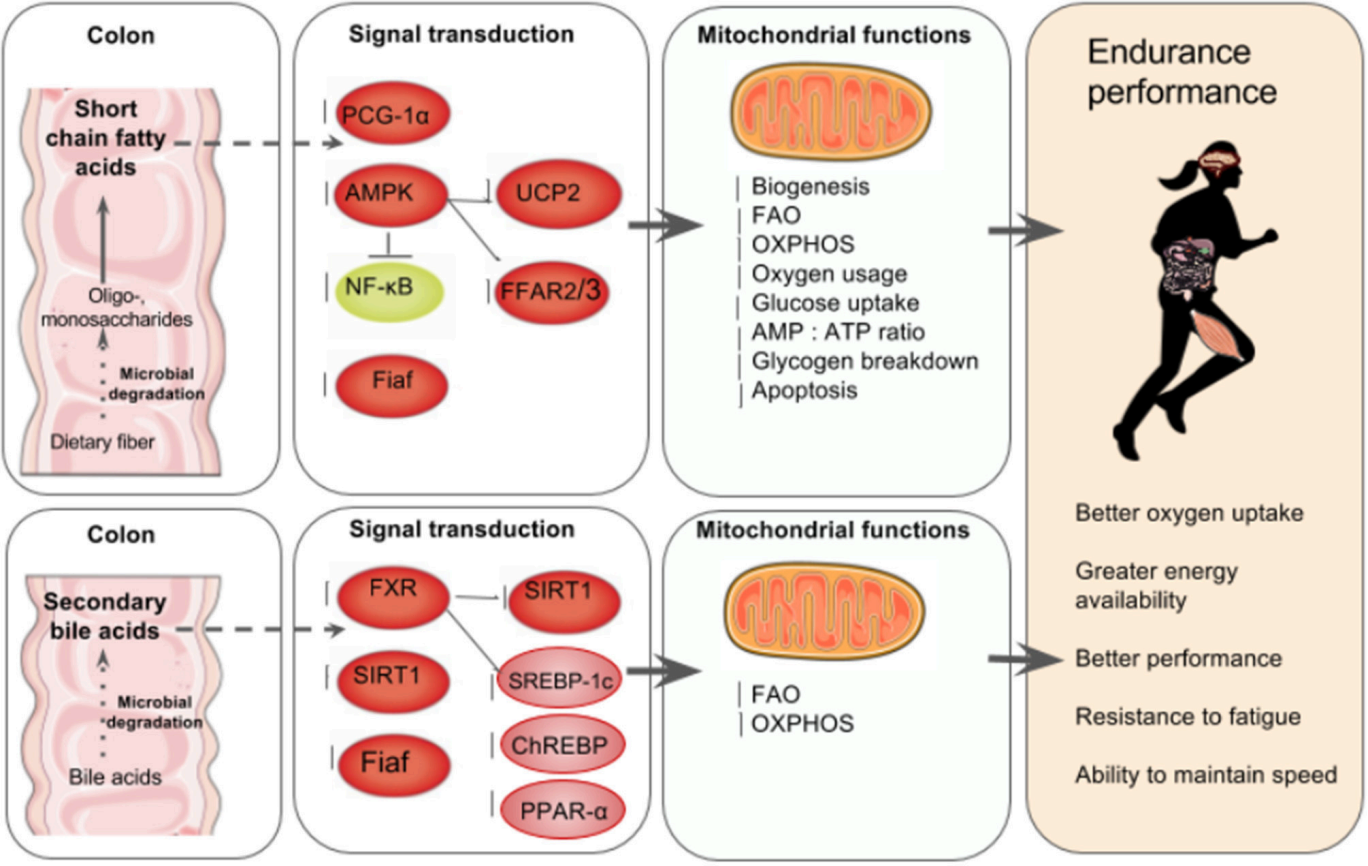
**The gut microbiota's regulation of mitochondrial energy production. Top left to right:** In the colon, the gut microbiota ferment indigestible dietary fiber such as resistant starch and oligosaccharides to produce SCFA in the intestines that can account for up to 10% of human caloric requirements (den Besten et al., 2013). SCFA are key mediators of mitochondria energy metabolism and act as ligands for free fatty acid receptors 2 and 3 (*FFAR2, FFAR3*) that regulate glucose and fatty acid metabolism (den Besten et al., 2013; Kimura et al., 2014). SCFA regulate SIRT1 which plays a role in mitochondrial biogenesis via *PGC-1*α deacetylation, (Lakhan and Kirchgessner, 2010; Radak et al., 2013). In skeletal muscle cells, butyrate phosphorylates AMPK and p38 which then activates *PGC-1*α and thus FAO and ATP production. Butyrate also activates AMPK via UCP2-AMPK-ACC pathway (den Besten et al., 2015). Commensal bacteria such as *Lactobacillus rhamnosus* CNCMI–4317 has been associated with increased *Fiaf* expression (Jacouton et al., 2015). In lamina propia macrophages, SCFA also inhibit NF-κB activation that reducing inflammation associated with ulcerative colitis (Lührs et al., 2002). The result is increased mitochondrial biogenesis, FAO, OXPHOS, oxygen usage, glucose uptake, AMP, ATP ratio and glycogen breakdown and reduced apoptosis (Lantier et al., 2014; Canfora et al., 2015; den Besten et al., 2015). **Bottom left to right:** Anaerobic bacteria degrade 5–10% of bile acids (Gérard, 2013), and secondary bile acids regulate carbohydrate and lipid metabolism by modulating the transcription factor receptors farnesoid X receptor (*FXR*) and G-coupled membrane protein 5 (*TGR5*) resulting is increased FAO and OXPHOS (Nie et al., 2015). FXR mediates carbohydrate metabolism via regulating *SIRT1* and *Fiaf* expression as well as *SREBP-1c* and *ChREBP* activation (Kuipers et al., 2014; Joyce and Gahn, 2016) and fatty acid metabolism via PPAR-α activation (Joyce and Gahn, 2016). There is increasing evidence that secondary bile acid metabolism might also directly modify mitochondrial biogenesis, inflammation and intestinal barrier function in different types of cells (Gao et al., 2009; Korecka et al., 2013; Alex et al., 2014; Caron et al., 2014; Kazgan et al., 2014). The result of SCFA and secondary bile acid's role in mitochondrial biogenesis is better overall athletic performance due to better oxygen uptake, energy availability and fatigue resistance.

Mitochondrial biogenesis occurs via fusion, which is the merging of mitochondria, or fission, which is the separation of damaged mitochondria (Busquets-Cortés et al., 2016). The greater number of mitochondria found in trained athletes' muscles enable better FAO, OXPHOS, and oxygen usage, which spares carbohydrate oxidation and thus glycogen breakdown and lowers lactate production (Hood et al., 2011; Rivera-Brown and Frontera, 2012). Conversely, a decrease in the number of mitochondria is related to impaired OXPHOS and FAO capacity (Wai and Langer, 2016). More than two in five marathon runners report experiencing a rapid onset of fatigue and the inability to maintain the speed and intensity of performance due to carbohydrate depletion (Rapoport, 2010) suggesting that poor OXPHOS and FAO capacity could be a major underlying cause of fatigue in athletes (Figure 2).

Given the energy requirements during endurance exercise, and the recently described complex and reciprocal relationship between the gut microbiota and whole body energy metabolism, it is not surprising that efforts to identify the mechanisms in which gut microbiota enhance mitochondrial FAO and OXPHOS in elite athletes are increasing (Pyne et al., 2015).

The gut microbiota contains more than 100 trillion microorganisms (Rajilić-Stojanović and de Vos, 2014), which comprise approximately 160 species and 9 million genes (Li et al., 2014) which are key to host energy metabolism. In the gut, anaerobic bacteria ferment and extract energy from otherwise indigestible polysaccharides such as fiber and resistant starch, and synthesize the byproduct SCFA such as N-butyrate, propionate and acetate (Flint et al., 2008). High fiber diets lead to 400–600 mmol of SCFA in the cecum per day, which accounts for approximately 10% of human caloric requirements (den Besten et al., 2013). In colonocytes, butyrate is transported to mitochondria where it undergoes FAO in aerobic conditions and becomes acetyl-CoA, which enters the Krebs cycle resulting in NADH which then enters into the electron transport chain producing ATP production and CO_2_ (Donohoe et al., 2011). Furthermore, butyrate is oxidized in mitochondria by a series of five enzymes including the mitochondrial enzyme acetoacetyl CoA thiolase, and butyrate oxidation has been shown to be impaired in ulcerative colitis patients (Roediger et al., 1993; Ahmad et al., 2000; Santhanam et al., 2007) suggesting that butyrate plays an important role in TCA activity in colonocytes and therefore overall colon health. Additionally, propionate and acetate can be carried into the bloodstream to various organs where they are used as substrates for mitochondrial oxidation, lipid production, and gluconeogenesis, which is synthesized from propionate (Nicholson et al., 2012).

Though human studies are lacking, various animal studies using germ-free (GF) animals or specific pathogen-free (SPF) animals have shown that the SCFAs, such as N-butyrate and acetate, may affect mitochondrial energy metabolism through a vast range of transcription factors that directly or indirectly control mitochondrial functions. The types and amount of SCFAs produced by gut microorganisms depends on the composition of the gut microbiota, metabolic interactions between microbial species and the amount and type of the main dietary macro- and micronutrients ingested (den Besten et al., 2013). The more plant-derived polysaccharides, oligosaccharides, resistant starch and dietary fiber one eats, the more these bacteria can ferment these indigestible food sources into beneficial SCFA.

Studies in C57BL/6 mice have shown that a sodium butyrate injection of 5% wt/wt in addition to a high fat diet (58% calories from fat) prevented insulin resistance by stimulating thermogenesis and fatty acid oxidation in skeletal muscle and brown adipose tissue mitochondria in part due to increased *PGC-1*α gene expression and protein activity (Gao et al., 2009). Interestingly, butyrate-mediated *PGC-1*α induction led to a transformation of skeletal muscle fibers from type II (glycolytic) to type I (oxidative), which are rich in mitochondria and stimulate FAO for ATP production (Gao et al., 2009). Similarly, rats fed human milk compared to cow or donkey milk displayed higher mitochondria energy efficiency associated with changes in the microbiota's capacity to produce N-butyrate (Trinchese et al., 2015). Therefore, it is interesting to speculate that N-butyrate could play an important role in *PGC-1*α activation, which is a biomarker of mitochondrial functions during endurance as *PGC-1*α gene expression has been shown to dramatically increase in skeletal muscles in response to exercise (Pilegaard et al., 2003; Bo et al., 2010; Little et al., 2010; Safdar et al., 2011).

N-butyrate and acetate may also affect mitochondrial function and metabolism via AMPK activation in colonocytes (Canfora et al., 2015; den Besten et al., 2015). AMPK has been shown to be a key energy sensor in skeletal muscles capable of regulating mitochondrial OXPHOS (Lantier et al., 2014). den Besten et al. (2015) discovered that SCFA activated the UCP2-AMPK-acetyl-CoA carboxylase (ACC) pathway which down regulated *PPAR*γ gene expression resulting in decreased lipogenesis and increased AMP: ATP ratio. The increased AMP: ATP ratio can also activate AMPK in liver, adipose (Richter and Ruderman, 2009) and muscle tissues (Hood et al., 2011), which stimulates glucose uptake, mitochondria FAO and OXPHOS and decreases protein and lipid synthesis. Similarly, Mollica et al. (2017) discovered that N-butyrate improved respiratory capacity and FAO through the activation of the AMPK-ACC pathway, which promoted mitochondrial fusion in liver and reduced insulin resistance and fat accumulation in obese mice. SCFA are also key mediators of mitochondria energy metabolism because they serve as a ligand for free fatty acid receptors 2 and 3 (*FFAR2, FFAR3*), also known as G-coupled receptor protein 43 (*GRP43*) and *GRP41* respectively, that regulate glucose and fatty acid metabolism (reviewed extensively by den Besten et al., 2013; Kimura et al., 2014), as well as *GPR109A* that activates pathways associated with energy homeostasis, lipid storage and food ingestion (Nicholson et al., 2012).

The impact of microbiota on mitochondrial functions has been further supported by studies intending to manipulate of gut microbiota through the use of probiotics. Administration of the probiotic *Lactobacillus rhamnosus* CNCMI–4317 was associated with greater *Fiaf* expression, also known as *ANGTPL4* (angiopoietin-like 4), through *PPAR-*α dependent pathways (Jacouton et al., 2015) that modified the OXPHOS capacity of mitochondria. In line with this, Bäckhed et al. (2007) demonstrated that GF mice expressed a lean phenotype because they were protected from the obesogenic effects of a high fat and sugar Western diet due to elevated *Fiaf* expression in the intestines, as well as increased AMPK and *PGC-1*α expression in skeletal muscles and the liver, which regulate mitochondrial OXPHOS in muscle cells (Lantier et al., 2014). Finally, certain intestinal bacteria such as *Eubacterium hallii* and *Anaerostipes caccae* have the capacity to transform the byproduct of anaerobic glycolysis lactate into SCFA during glucose depletion thus creating an alternative energy source for the host (Duncan et al., 2004; Scott et al., 2013) while bypassing OXPHOS (Rogatzki et al., 2015).

Besides SCFA, secondary bile acids produced by the gut microbiota also play an important role in regulating mitochondrial energy metabolism. Anaerobic bacteria of the genera *Bacteroides, Eubacterium*, and *Clostridium* degrade 5–10% of the primary bile acids forming secondary bile acids (Gérard, 2013). Secondary bile acids interact with mitochondria by modulating transcription factors related to lipid and carbohydrate metabolism, including farnesoid X receptor (*FXR*) and G-coupled membrane protein 5 (*TGR5*) (Nie et al., 2015). *FXR* is a target of NAD-dependent protein deacetylase *SIRT1* (reviewed by Kuipers et al., 2014) and regulates the steroid response element binding protein-1c (*SREBP-1c*), carbohydrate response element binding protein (*ChREBP*), and *PPAR-*α, which stimulates fatty acid uptake and oxidation (Joyce and Gahn, 2016).

There is increasing evidence that secondary bile acid metabolism might also directly modify *SIRT1* and *Fiaf* expression as well as mitochondrial biogenesis, inflammation and intestinal barrier function in different types of cells (Gao et al., 2009; Korecka et al., 2013; Alex et al., 2014; Caron et al., 2014; Kazgan et al., 2014). *SIRT1*'s role in gut homeostasis is also beginning to be elucidated shedding new light on the role gut microbiota-mitochondria crosstalk plays in energy metabolism (Kazgan et al., 2014; Lo Sasso et al., 2014). Because 12 weeks of voluntary running wheel in wild type mice enhanced biliary bile acid secretion and increased fecal bile acid and neutral sterol outputs compared to sedentary controls (Meissner et al., 2011), it is temping to speculate about the role of microbiota plays in energy metabolism through the modulation of bile acid, SCFA and indirect induction of *SIRT1, Fiaf* and *FXR* genes (Figure 2).

Unlike the beneficial effects commensal bacteria have on energy metabolism, pathogens such as *Salmonella* and *Escherichia coli* (Leschelle et al., 2005) can produce negative effects for the host mitochondria energy metabolism by degrading sulfur amino acids to produce hydrogen sulfide (H_2_S) in the large intestines. H_2_S is an important mediator of many physiological and pathological processes. High amounts of H_2_S can inhibit a key component of the mitochondrial respiratory chain by penetrating cell membranes and inhibiting COX activity and energy production (Blachier et al., 2007; Mottawea et al., 2016). Pathobionts can also produce NO, which may affect host mitochondrial activity and favor bacterial infection (Vermeiren et al., 2012). Besides pathobionts, high protein diets, which are common in many elite athletes, can result in high levels of H_2_S and urea in the gut (reviewed by Windey et al., 2012). For example, and fecal H_2_S concentrations can reach up to 3.4 mM upon eating a high protein diet in humans (Leschelle et al., 2005). The high concentrations of gut-derived H_2_S treatment led to decreased COX expression in human colonic HT-29 cells and thus reduced electron transfer of complexes I and II in the respiratory chain shifting oxidative metabolism toward glycolysis, increased lactate production and decreased ATP production (Leschelle et al., 2005). Similarly, Beaumont et al. (2016) concluded that exposure of high levels of H_2_S to HT-29 human cells showed not only reduced mitochondrial oxygen consumption but also an increase in the expression of inflammatory genes such as IL-6, which was increased following a high protein diet. Mottawea et al. (2016) recently demonstrated that a proliferation of pathobionts, many of which are known to be potent H_2_S producers, down regulated mitochondrial proteins.

Additionally, H_2_S can inhibit butyrate β-oxidation in the colon, which is believed to contribute to ulcerative colitis (Leschelle et al., 2005; Blachier et al., 2007). In line with this, Le Chatelier et al. (2013) studied microbial diversity in obese vs. healthy individuals. They discovered that those individuals who had high bacterial diversity had higher hydrogen and organic acid production (i.e., N-butyrate, propionate) whereas those with low bacterial richness had less N-butyrate- producing bacteria, increased H_2_S forming potential and *Campylobacter/Shigella* abundance and an overall inflammation-associated microbiota.

Given the lack of studies in human endurance athletes, it's difficult to make precise dietary recommendations for endurance athletes in regards to how to optimize SCFA and bile acid metabolism for energy production. However, high animal protein consumption during resting days and training may negatively affect the gut microbiota of elite athletes (e.g., production of potentially toxic byproducts such as H_2_S). It is clear then that the interaction between diet and exercise needs to be further studied in order to better assess the contributions of diet and microbial activities in mitochondria functions and athletic performance.

#### The gut microbiota's regulation of mitochondrial ROS production

During and after exercise, ROS, and RONS production increases in response to energy needs and are primarily produced from electron leak in the mitochondrial electron transport chain (Radak et al., 2013). Complex I of the mitochondrial electron transport chain is one of the greatest generators of ROS, RONS, and free radicals such as NO and superoxide anion (${\text{O}}_{2}^{-}$), which are molecules that are unstable because they possess an impaired electron that causes oxidation reactions with proteins, lipids and DNA in order to become stable molecules (Fisher-Wellman and Bloomer, 2009; Hood et al., 2011; Gomes et al., 2012; Radak et al., 2013).

Exercise-induced ROS production during regular physical activity can lead to beneficial adaptations to exercise such as vasoregulation, fibroblast proliferation (Gomes et al., 2012), muscle hypertrophy, increased mitochondrial biogenesis, induction of antioxidant enzymes (Radak et al., 2013) and regulation of immune responses to eliminate antigens (Fisher-Wellman and Bloomer, 2009; Radak et al., 2013). Ten days of endurance exercise can elevate antioxidant production and decrease inflammation resulting in less intestinal permeability (Holland et al., 2015). The generally accepted hypothesis is that mitochondrial ROS can induce NF-κβ and activator protein 1 (AP-1) which activates antioxidant enzymes such as manganese superoxide dismutase (Mn-SOD) (Radak et al., 2013), catalase and SOD (Blaser et al., 2016), as well as *PGC-1*α (Steinberg et al., 2006), thereby having a homeostatic effect (Blaser et al., 2016). In *PGC-1*α knockout mice, St-Pierre et al. (2006) observed a decrease of Mn-SOD, copper–zinc superoxide dismutase levels, suggesting that *PGC-1*α knockout mice are more sensitive to oxidative stress (St-Pierre et al., 2006).

**Figure 3:**
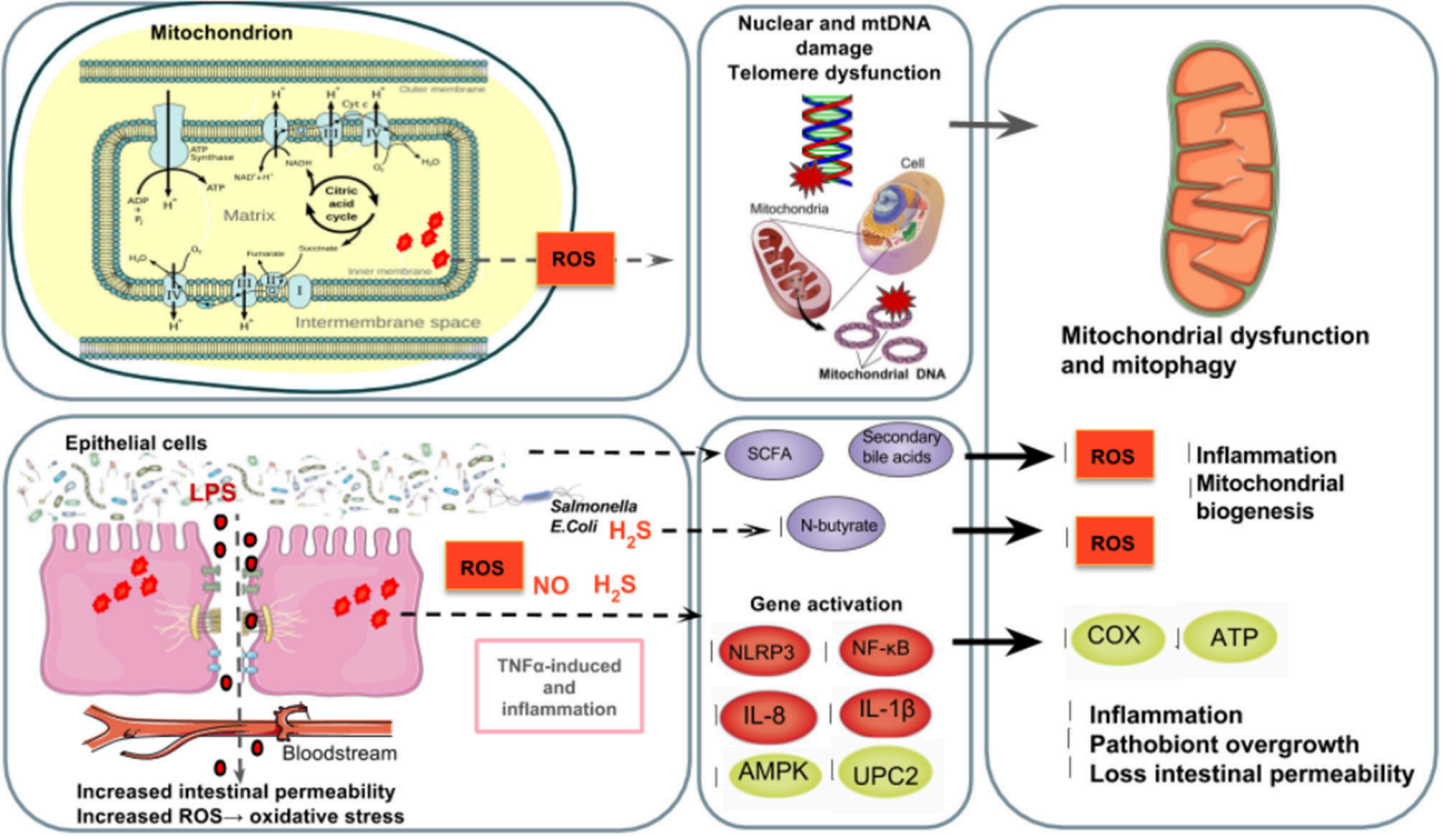
**The gut microbiota's regulation of mitochondrial ROS production. Top left to right:** Athletes have two major sources of ROS and RONS: the mitochondrial electron transport chain and the intestinal epithelial cells and transmigrating neutrophils in the gut lumen (Holland et al., 2015) in which free radicals such as NO and superoxide are produced (Fisher-Wellman and Bloomer, 2009; Gomes et al., 2012; Radak et al., 2013). Poorly trained individuals and athletes who overtrain are at a higher risk of suffering from oxidative stress (Radak et al., 2008) causing ROS-induced DNA (Radak et al., 2013), which increases mutations in DNA (Green et al., 2011), shortens telomere length (Wallace et al., 2010) and alters mitochondrial biogenesis (Sahin et al., 2011). **Bottom left to right:** The excessive release of stress hormones overtrained athletes experience as well as increased body oxygen uptake can generate ROS and RONS in the tissues that undergo ischemia and hypoperfusion (Mach et al., 2017). Ischemia-induced intestinal hyperpermeability (Holland et al., 2015) can induce LPS translocation and an inflammatory cascade of TNFα (Clark and Mach, 2016), the ROS-triggering OXPHOS inhibitor and inflammasome NLRP3 which results in a mitochondria-mediated inflammatory responses (Green et al., 2011; de Zoete and Flavell, 2013) and mitophagy (Shimada et al., 2012), as well as NF-κB, IL-1β, IL- 6, and IL-8 expression (Liu et al., 2012). TNFα and IL-6 inhibit AMPK activation, which reduces glucose metabolism and FAO in mitochondria (Steinberg et al., 2006; Lim et al., 2010; Viollet et al., 2010; Andreasen et al., 2011). Reduced expression of uncoupling protein 2 (UPC2) can lead to partial uncoupling of mitochondrial OXPHOS (Crouser et al., 2002) and elevated ROS production (Saint-Georges-Chaumet et al., 2015). Furthermore, pathobionts (i.e., *Fusobacterium, Veillonella*, and *Atopobium parvulum*) can produce hydrogen sulfide (H_2_S) and nitrogen oxide (NO) which favors infectious proliferation and inflammation (Mottawea et al., 2016), inhibition of COX activity and butyrate β-oxidation in the colon (Leschelle et al., 2005; Blachier et al., 2007) which negatively affects mitochondrial function and energy production (Blachier et al., 2007; Mottawea et al., 2016). On the other hand, SCFA such as N-butyrate and secondary bile acids, might influence mitochondrial functions related to energy production, mitochondrial biogenesis, redox balance and inflammatory cascades, making it a potential therapeutic target for endurance (Circu and Aw, 2012; Bär et al., 2013; den Besten et al., 2013; Mottawea et al., 2016).

However, many athletes suffer from stress and enter into a vicious cycle of over exerting themselves with strenuous training and competitions, which in chronically high levels of ROS and RONS which can deplete the non-enzymatic antioxidant system and damage cellular function. In over trained athletes, the excessive release of stress hormones induced by physical as well as increased body oxygen uptake, might lead to the generation of ROS and RONS in the tissues that undergo ischemia and hypoperfusion (Mach et al., 2017). Ischemia-induced intestinal hyperpermeability typically occurs in humans exercising at 70% maximal oxygen consumption when blood supply is reduced by at least 50% (Holland et al., 2015). Therefore, athletes have two major sources of ROS and RONS: from the electron transport chain in mitochondria and the intestines by both epithelial cells and transmigrating neutrophils in the gut lumen (Figure 3).

It appears that both poorly trained individuals and athletes who overtrain are at a higher risk of suffering from oxidative stress (Radak et al., 2008), which is characterized by a substantial increase in ROS damage of lipids, proteins, and DNA (Hood et al., 2011; Radak et al., 2013). DNA oxidative damage increases the ratio of mutations in mitochondrial and nucleus genome (Green et al., 2011). Importantly, mitochondrial DNA is more susceptible to oxidative damage and mutation accumulation due to its proximity to ROS produced in mitochondria (Lee and Wei, 2005; Crane et al., 2013). Additionally, recent studies have revealed that ROS production also reduces telomere length while modifying mitochondrial biogenesis (Sahin et al., 2011). Telomere dysfunction activates p53-mediated pathway to repress the expression of *PPAR-*γ, *PGC-1*α, and *PGC-1* (Sahin and DePinho, 2012). In turn, the repression of both co-activators impairs functional mitochondrial biogenesis leading to higher levels of ROS damaging both telomere and mitochondrial DNA, and starts a negative feedback loop. Moreover, several studies reported lately that positive correlations have been observed between telomere length and mitochondrial DNA copy number variations in healthy adults (Kim et al., 2013) and between telomere length and aging and several diseases (Garatachea et al., 2015).

Palazzetti et al. (2003) analyzed oxidative stress levels in male triathletes vs. sedentary individuals before and after a 4-week period of overtraining through blood and urine samples. They discovered that overtraining could compromise the antioxidant defense mechanism due to increased lipid peroxidation and an up regulation of plasma glutathione peroxidase (GSH-Px), which failed to prevent oxidative damage, which is in line with other studies in athletes who overtrain (Palezzetti et al., 2003). Bloomer et al. (2005) also showed that just 30 min of acute anaerobic and aerobic exercise can also lead to redox imbalance as demonstrated by a significant increase in glutathione oxidation (decreased GSH and increased GSSG post-exercise), increased lipid peroxidation (protein carbonyls) and minor increase in DNA oxidation (8-OHdG). In mice, Li et al. (2015) reported that acute exercise (76% VO_2_ max for 15, 60, or 90 min) induced mitochondrial oxidative stress. This was confirmed by increased ROS production, which induced the inflammasome-NOD-like receptor family and pyrin domain containing 3 (*NLRP3*). Similarly, we have recently demonstrated in horses, which are considered to serve as an optimal *in vivo* model for characterizing the response to endurance exercise, that the elevated respiration rates during endurance exercise had led to the generation of more ROS than the antioxidant systems can scavenge (Mach et al., 2016, 2017). Of note, many studies in athletes have varying results on oxidative markers due to the heterogeneity of methodology, blood, urine and DNA analysis, athlete training status, type of exercise, exercise conditions, long-term effects of overtraining and athlete diet. Therefore, it is difficult to make generalizations about oxidative status pre and post-exercise in endurance athletes; however, it appears that in general, well-trained athletes who train 20–30 h per week likely have lower RONS production and better antioxidant defense mechanisms and thus redox balance (reviewed by Fisher-Wellman and Bloomer, 2009).

In the last years there has been a proliferation of experimental works, conducted mainly in animals, aimed to explore how the microbiota modulates mitochondrial ROS production. As mentioned above, evidence indicates that gut microbiota communicate with mitochondria via SCFA, which have antioxidative properties. The absence of microbial colonization in mice was associated to a reduced levels of SCFA and serum levels of GPx and catalase after endurance swimming, which are critical antioxidants for reducing oxidative stress (Hsu et al., 2015). Different studies using mice models (Dobashi et al., 2011, 2013) have reported that the gut microbiota regulate SOD activity, yet more studies are needed to better understand the gut microbiota's role in controlling the intestinal redox balance in response to long-term intense exercise, inflammation and dysbiosis.

Other studies have shown that SCFA may inhibit telomere length shortening (Garatachea et al., 2015). O'Callaghan et al. (2012) discovered that higher colonic SCFA production in rats was associated with reduced malondialdehyde levels (a marker of oxidative stress), telomere shortening and DNA damage. Though the exact mechanisms of how SCFA reduced telomere damage in this experiment are unknown, other studies have shown that N-butyrate possess antioxidant properties that can reduce H_2_${\text{O}}_{2}^{-}$ induced DNA damage possibly by altering chromatin structures such as telomere length, and induce glutathione antioxidant production (Rosignoli et al., 2001; Hamer et al., 2009). N-butyrate can reduce colonic H_2_O_2_ levels and thus diminish oxidative damage by increasing COX-2 activity (Martinez et al., 2010). In summary, SCFA can improve mitochondrial function by reducing ROS levels through various possible mechanisms which can damage DNA and shorten telomeres which are both associated with aging and mitochondrial dysfunction.

The gut microbiota can also metabolize the aromatic amino acid tryptophan, which apart from being the precursor to the neurotransmitter serotonin also plays a role in synthesizing the cofactors for redox reactions nicotinamide adenine dinucleotide (NAD) and NAD phosphate (NADP) (Ito et al., 2003; Richard et al., 2009). Other bacteria might use tryptophan to produce quinolinic acid, which is a neuroactive metabolite of the kynurenine pathway often implicated in the pathogenesis of a variety of human neurological diseases and lipid peroxidation (Kurnasov et al., 2003). A comparative genomic analysis proved that several bacteria contain bacterial enzymes such as 3-hydroxy-kynureninase, necessary to convert tryptophan to quinolinic acid (Kurnasov et al., 2003). *Strephtomyces antibioticus, Cyanidium caldarium, Karlingia rosea*, and *Xanthomonas pruni* use tryptophan to biosynthesize quinolinic acid, and *Pseudomonas aureofaciens* have enzymatic activities such as tryptophan dioxygenase and kynureninase (Kurnasov et al., 2003). Other pathobionts such as *Bacteroides thetaiotaomicron, Proteus vulgaris* and *E. coli* possess the enzyme bacterial tryptophase, which metabolizes tryptophan into metabolites such as indole-3-pyruvate, indole-3-lactate, and indole-3-acetate (Boulangé et al., 2016). Indole-3-pyruvate can be converted downstream into indole-3-acetate, which can activate horseradish peroxides causing free radical formation and lipid peroxidation (Kumavath et al., 2017). Lastly, some intestinal pathobionts have adapted regulatory responses to oxidative stress (Chiang and Schellhorn, 2012) that can lead to either supports the growth of pathogens or inhibits N-butyrate production (Rivera-Chávez et al., 2017). For instance, the increased colonic nitrate content favors the proliferation of *Enterobacteriaceae* pathogens such as *E. coli* and *Salmonella* spp. through nitrate respiration (Rivera-Chávez et al., 2017; Zeng et al., 2017). These results illustrate that non-commensal bacteria could induce redox imbalance and inflammatory pathways in the gut.

Ghosh et al. (2011) believe that ROS production is a defense mechanism that can elicit cytotoxicity against the pathogen and reduce the burden of infection on the host. In fact, Ghosh et al. (2011) showed that *Citrobacter rodentium* infection in C57BL/6 resistant resulted in more Bacteroides and elevated levels of oxidative stress (reduced GSH ratio, induction of iNOS, and reduced antioxidant MnSOD/SOD2 protein expressions). A review by Lobet et al. (2015) describes how ROS production have been involved in the clearance of different intracellular pathogens such as *Listeria monocytogenes, Staphilococcus typhimurium*, or *Toxoplasma gondii*. However, it has been shown that in order to overcome the mitochondrial effect on the immune response and cell survival, numerous bacterial species of microbiota tend to directly reduce mitochondrial ROS production (Lobet et al., 2015). For instance, *Mycobacterium tuberculosis* downregulates LPS-induced TLR signaling pathways that reduce mitochondrial ROS production (Saint-Georges-Chaumet and Edeas, 2016). Other microbial toxins can upregulate activity of the detoxification enzyme mitochondrial superoxide dismutase, which results in a lower ROS content and reduces host cell apoptosis, as observed in *Ehrlichia chaffeensis* (Liu et al., 2012).

#### The gut microbiota's regulation of mitochondrial inflammatory activity

As reviewed by Mach and Fuster-Botella (in press), intense exercise raises plasma cortisol levels inducing an influx of neutrophils from bone marrow and in efflux of other leukocyte subsets, which causes an acute-phase inflammatory response. In contrast to habitual light exercise and fitness (Clarke et al., 2014), strenuous exercise causes an increase in the number of pro-inflammatory cytokines, such as TNFα, IL-1, IL-6, IL-1 receptor antagonist, TNF receptors, as well as anti-inflammatory modulators like IL-10, IL-8, and macrophage inflammatory protein-1, indicating a dose-response effect between biological responses to exercise and host immunity (reviewed by Clark and Mach, 2016).

A key mechanism of inflammation is the activation of inflammasomes, which are large cytosolic protein complexes that activate caspase-1, which induce the inflammatory cytokines IL-1 and IL-18 and facilitates other inflammatory mediators that are crucial for the innate immune response. Mitochondria play a central role in the initiation of inflammasomes and other inflammatory pathways and as such integrate autophagy, cell death, and inflammation (Green et al., 2011). Although the exact mechanisms are currently unknown, noninfectious agents such as ROS-triggering OXPHOS inhibitors and pro-inflammatory signals seems to activate *NLRP3*, which results in a mitochondria-mediated inflammatory responses (Green et al., 2011; de Zoete and Flavell, 2013). The activation of NLRP3 also promotes mitophagy (mitochondrial autophagy), a cellular process that eliminates malfunctioning mitochondria (Shimada et al., 2012). As previously mentioned, Li et al. (2015) reported that mice that performed acute exercise (76% VO_2_ max for 15, 60, or 90 min) experienced exercise-induced mitochondrial stress, which was confirmed by increased ROS production and *NLRP3* expression.

Another way endurance training can cause changes in immune responses and mitochondrial function is by reducing the gastrointestinal blood flow, oxygen and nutrients while increasing tissue hyperthermia, permeability of the gastrointestinal epithelial wall and the destruction of gut mucous thickness (Marlicz and Loniewski, 2015), which stimulates an inflammatory immune response. These processes are associated with cell damage and lipopolysaccharides (LPS) translocation outside of the gastrointestinal tract, which consequently triggers immune and inflammatory responses often resulting in increased intestinal permeability (reviewed by Clark and Mach, 2016) but also mitochondria functions. For example, studies in animal models have shown that LPS injection (3 mg/kg) in adult male cats resulted in a partial uncoupling of mitochondrial OXPHOS and a 40% reduction of COX activity (Crouser et al., 2002), but also to higher ROS production as a result of a reduction in the expression of uncoupling protein 2 (*UPC2*; Saint-Georges-Chaumet et al., 2015). Lee and Hüttemann (2014) postulated that TLRs that bind to LPS stimulate TNFα and IL-6 in production, which activates tyrosine kinase leading to downstream COX phosphorylation and impaired ATP production in mitochondria.

On the other hand, evidence is emerging that *SIRT1* activity can attenuate LPS- induced inflammatory cytokines and signaling while improving mitochondrial functions (Caruso et al., 2014; Lo Sasso et al., 2014; Wei et al., 2017). Cheng et al. (2015) revealed that exercise activated *SIRT1* activity, which reduced NF-κB, TLR-4, IL-1β, IL- 6, IL-8, and TNFα inflammatory activity. A murine sepsis model in C57BL/6 mice injected with LPS stimulated TLR4 and activated *SIRT1*, which induced NF-κB p65 deacetylation and deactivation thus inhibiting pro-inflammatory gene expression (Liu et al., 2012). SIRT1 could play an important role in maintaining intestinal barrier function through various mechanisms such as enhancing crypt proliferation and suppressing villous apoptosis (Wang et al., 2012), stimulating intestinal stem cell expansion in the gut (Igarashi and Guarente, 2016), regulating tight junction expression of zonulin ocludin-1, occluding, and claudin-1 during hypoxia (Ma Y. et al., 2014). SIRT1 can also reduce stress-induced inflammation and can improve intestinal ischemia/reperfusion-induced ROS accumulation and apoptosis via miR-34a-5p activation, which induces SIRT1-mediated suppression of intestinal ROS accumulation (Wang et al., 2016). However, some studies have shown that LPS-induced inflammatory signaling can inhibit *SIRT1* activity (Shen et al., 2009; Fernandes et al., 2012; Storka et al., 2013) suggesting that *SIRT1* activity or inactivity may be tissue specific and dependent upon the dose of LPS.

Other studies show that inflammatory cytokines such as TNFα and IL-6 can also inhibit AMPK activation, which negatively affects glucose metabolism and reduces FAO in mitochondria (Steinberg et al., 2006; Lim et al., 2010; Viollet et al., 2010; Andreasen et al., 2011). IL-6 which is another cytokine associated with exercise-induced muscle injury (Richter and Ruderman, 2009; Lantier et al., 2014) and intestinal permeability (Grootjans et al., 2010) can also activate AMPK upon prolonged exercise in skeletal muscle (Febbraio and Pedersen, 2005; Ruderman et al., 2006), which can improve peripheral glucose uptake and whole body insulin sensitivity (Glund et al., 2007). Furthermore, IL-6 synthesis in skeletal muscles can increase up to 100-fold in marathon runners (Febbraio and Pedersen, 2002). Ruderman et al. (2006) tested the metabolic effects IL-6 had on AMPK activation during exercise in IL-6 knockout mice. They concluded that mature 9-month old mice showed signs of dyslipidemia, impaired glucose tolerance and obesity possibly due to the lack of IL-6-induced activation of AMPK. Kelly et al. (2009) analyzed IL-6- induced AMPK activation in rat skeletal muscle cells *in vivo* injected with 25 ng/g animal weight. They discovered that IL-6 activated AMPK by increasing cyclic adenosine monophosphate (cAMP) concentration and the AMP: ATP ratio leading to energy synthesis in muscles. Another study by Lantier et al. (2014) showed that AMPKα1α2 knockout mice had a significantly reduced exercise performance and fatigue resistance as well as increased IL-6 expression possibly due to skeletal muscle injury. This study clearly provides evidence that AMPK inactivity reduced mitochondrial OXPHOS that resulted in reduced energy production in response to exercise.

Subproducts of commensal microbiota may also regulate mitochondrial inflammatory responses through different mechanisms during endurance exercise, the principal one likely being modulating the intestinal barrier-ROS production and LPS translocation. For example, bacteria-derived indole-3-propionate has beneficial effects for the host because it maintains intestinal barrier function by up regulating tight junction proteins and downregulating TNFα in enterocytes, which prevents the translocation of LPS and other pathogens thus reducing inflammatory immune responses (Boulangé et al., 2016). SCFA also downregulate LPS- induced pro-inflammatory mediators via histone decetylation in macrophages in the lamina propria as well as pro-inflammatory mediators such as NO, IL-6, and IL-12 (Chang et al., 2014). Additionally, SCFA inhibit NF-κB activation in lamina propia macrophages that reduced inflammation that is associated with ulcerative colitis (Lührs et al., 2002) and can also regulate AMPK (Canfora et al., 2015) by activating the UCP2-AMPK-acetyl-CoA carboxylase (ACC) pathway (den Besten et al., 2015) as well as the GPR43 protein, which play a protective role in the inflammatory activation and signaling. For example, Peng et al. (2009) determined that human colonic epithelial cell line Caco-2 treated with 2 mmol/L of N-butyrate presented an upregulation of AMPK activity, together with an increased transepithelial resistance, a marker of proper intestinal barrier function. Yet AMPK's activation and role in tight junction protein regulation remain unknown, though it's likely via phosphorylation (Elamin et al., 2013). Vieira et al. (2015) discovered that when the metabolic sensor receptor *GPR43* sensed acetate in neutrophils *in vitro*, IL-18 production increased, which may promote gut epithelial integrity in colitis models (Zaki et al., 2010; Macia et al., 2015). Similarly, Macia et al. (2015) reported that diets rich in fibers increased the colonic acetate in mice that suffered DSS-induced colitis, and consequently increased the *GPR43* expression and the IL-18 secretion. On the other hand, a diet that increased ketone metabolite β-hydroxybutyrate levels inhibited *NLRP3* activity during intense exercise (Shao et al., 2015; Youm et al., 2015).

However, the inflamed microenvironment in the gut might confer a favorable environment for the expansion of pathogenic *Enterobacteriaceae* (Zeng et al., 2017). *Enterobacteriacea*, including *E. coli, Klebsiella* spp., and *Proteus* spp. are among the most commonly overgrown pathobionts in inflammatory conditions (Zeng et al., 2017). Inflammation in the gut can cause an elevation in oxygen to the intestinal lumen as blood flow and hemoglobin increase and induce the growth of pathogens in the gut (Zeng et al., 2017). Other bacteria, such as *Chlamydia pneumonia* (Shimada et al., 2012) and *Salmonella typhimurium* can induce *NLRP3* via Caspase-11 macrophages (de Zoete and Flavell, 2013) and cause mitochondrial dysfunction.

### How mitochondria regulate the gut microbiota

Lobet et al. (2015) describe that mitochondria participate in the detection of infectious microorganisms and cellular damage to activate innate immune responses. Additionally, it has been demonstrated that mitochondria have a prominent role in the modulation of gut functions (Igarashi and Guarente, 2016; Wang et al., 2016), such as intestinal barrier protection (Peng et al., 2009) and mucosal immune response, all of which are important for the maintenance of the mucus layer (Ma Y. et al., 2014) and intestinal microbiota (Shimada et al., 2012; Caron et al., 2014). Thus, a dysregulation of mitochondrial functions can affect the gut microbiota through the perturbation of the normal intestinal habitat allowing bacterial antigens to penetrate the epithelium and stimulate the immune response. As previously explained, another possible perturbation in the microbiota habitat induced by mitochondria occurs through the modifications of immune system responses.

It has been shown that genetic variants in the mitochondrial genome might also regulate the gut microbiota. Until recently, mitochondrial genetics was often considered outside mainstream genetics. The polyploidy nature of the mitochondrial genome—up to several thousand copies per cell—gives rise to an important feature of mitochondrial genetics, homoplasmy, and heteroplasmy. Some mutations affect all copies of the mitochondrial genome (homoplasmic mutation), whereas others are only present in some copies of the mitochondrial genome (heteroplasmic mutation) (Taylor and Turnbull, 2005).

Polymorphisms in the *ND5*, and *CYTB* genes or D- Loop region of mitochondrial genome have been associated with specific gut microbiota compositions. For instance, Ma J. et al. (2014) demonstrated that A13434G, a synonymous SNP located on the *ND5* gene and the synonymous SNP T15784C located on *CYTB* were strongly associated with *Eubacterium* (belonging to *Clostridium* cluster IV) and *Roseburia* genera abundance (*Clostridium* cluster XIVa), which are highly oxygen-sensitive anaerobes and butyrate producers. Similarly, the non-synonymous polymorphism T14798C, which maps to cytochrome b gene, was strongly associated with differential abundance of *Dialister* in the vaginal posterior fornix (Evaldson et al., 1980). These findings suggest that the host mitochondrial genome variants might inherently define the gut microbiome composition and function, which in turn will structure their community.

Moreover, nuclear genome mutations that cause imbalances in mitochondrial functions (e.g., gene *TYMP* mutation that results in the patients with mitochondrial neuro gastro intestinal encephalomyopathy) or disorders in the long-chain fatty acid oxidation in the mitochondrion (e.g., carnitine palmitoyltransferase 1A deficiency biallelic pathogenic variants) are more prone to bacterial infection than general population (Garone et al., 2011; Gessner et al., 2013). Additionally, the European mitochondrial DNA haplogroup HV has been associated with decreased odds of severe sepsis, suggesting that mitochondrial haplotypes could determine survival rates during severe septic shock due to differences in its function (Baudouin et al., 2005; Lorente et al., 2012; Jiménez-Sousa et al., 2015), higher OXPHOS capacity as well as higher ROS and RONS production (Jiménez-Sousa et al., 2015). More specifically, Maruszak et al. (2014) reported that Olympic athletes were primarily from the HV haplogroup, which has been associated with a higher VO_2_ max in response to exercise coupled to more OXPHOS capacity and thus more aerobic ATP production, whereas a study in healthy male Spanish Caucasians (*n* = 81) also demonstrated that the HV haplogroup was associated with higher ROS production and mitochondrial oxidative damage compared to the JT haplogroup (Martinez et al., 2010).

Interestingly, mitochondria replicate during endurance exercise, so mitochondrial DNA might accumulate mutations that eventually compromise the efficiency of OXPHOS and other essential functions including intestinal homeostasis (Green et al., 2011). Although, Safdar et al. (2011) showed that endurance exercise reduced the frequency of point mutations in the PolG mice, resulting in a concomitant increase in mitochondrial COX complex assembly, mitochondrial heteroplasmy can easily occur in endurance athletes due to the fact that each cell contains between 1,000 and 100,000 copies of mitochondrial DNA (Khan et al., 2015), which in turn can be passed down to subsequent generations (Stewart and Chinnery, 2015). Ensuing cell divisions can give rise to either an increased or decreased frequency of a given mutation as well as de novo mutations during an individual's lifetime (Stewart and Chinnery, 2015). Genome-wide studies that attempt to characterize specific mitochondrial genes and pathways in the human nuclear and mitochondrial genome that shape the composition of the microbiome of endurance athletes are needed.

More studies are needed to understand how mitochondrial heteroplasmy and nuclear genetic variants affect mitochondrial functions and in turn, the microbiota composition and function. A holistic study that imposes to grasp the complex dynamics of the interaction between the environment, stochasticity and the three genomes (nuclear, mitochondrial and gut microbiome) is required.

## Conclusion

Many lines of evidence suggest that mitochondria have a central role in energy production, ROS and RONS production and regulation of inflammasomes during endurance exercise. Endurance exercise induces systemic mitochondrial biogenesis, prevents mitochondrial DNA depletion and mutations, and increases mitochondrial oxidative and antioxidant capacity. However, overtraining and chronic stress promotes inflammation in the gastrointestinal tract of athletes which results in a plethora of stressors that favor the lipopolysaccharide translocation and the proliferation of pathobionts. We are still in the infancy of understanding the bidirectional crosstalk between the gut microbiota and mitochondria, but several studies shown that commensal gut microbiota molecules, such as N-butyrate, are essential for controlling mitochondrial oxidative stress and inflammatory responses, pathogen growth and adherence as well as in improving metabolism and energy expenditure during exercise. Furthermore, short chain fatty acids can induce key mediators in mitochondrial biogenesis through transcriptional co-activators such as PCG-1α, the redox sensitive energy sensor *SIRT1* and the enzyme AMPK, which suppress a broad inflammatory response and mediate beneficial effects of exercise. Dampening inflammation and oxidative stress during endurance exercise would be an ideal approach to restricting blooms of pathobionts in the gut as well as their detrimental effects on mitochondrial functions. Although it remains a challenge to tone down inflammatory response through dietary treatments, maneuvering nutritional changes and oxidative stress might be a good approach to maintain a healthy gut microbiota and thereby maintain mitochondrial functions and host homeostasis. On the other hand, mitochondrial ROS production has a crucial role in the regulation of gut functions such as intestinal barrier integrity and mucosal immune responses, all of which are important for regulating the gut microbiota composition. Despite the diminutive size of the mitochondrial genome, mitochondrial DNA mutations are inherited and might affect not only tissue functions but also microbiota functions. Mutations in mitochondrial genes (i.e., *ND5*, and *CYTB*) or in the D- Loop region and oxidative stress also modulate gut microbiota composition and functions. Therefore, understanding the multiple molecular pathways that lead to mitochondrial DNA mutations accumulations is needed.

## Author contributions

AC wrote the main text and both AC and NM designed the figures. NM provided critical feedback on content, design and revision of the manuscript. Both authors have edited and approved the final version of the manuscript.

### Conflict of interest statement

The authors declare that the research was conducted in the absence of any commercial or financial relationships that could be construed as a potential conflict of interest. The reviewer MB and handling Editor declared their shared affiliation, and the handling Editor states that the process nevertheless met the standards of a fair and objective review.

## Abbreviations

8-OhdG: 8-hydroxy-2-deoxyguanosine
ACC: acetyl-CoA carboxylase
AP-1: activator protein-1
AMP: adenosine monophosphate
ATP: adenosine triphosphate
AMPK: 5′ adenosine monophosphate-activated protein kinase
ANGTPL4: angiopoietin-like 4
*atp*1: ATP synthase 1
*atp*3: ATP synthase 3
ChREBP: carbohydrate response element binding protein
cAMP: cyclic adenosine monophosphate
CREB: cyclic-AMP response element binding protein
COX: cytochrome c oxidase
FXR: farnesoid X receptor
FIAF: fasting-induced adipose factor
FAO: fatty acid β-oxidation
FFAR2: free fatty acid receptor 2
FFAR3: free fatty acid receptor 3
GPR: G coupled protein receptor
TGR5: G-coupled membrane protein 5
GF: germ free
GPx: glutathione peroxidase
GSH-Px: glutathione peroxidase
H_2_S: hydrogen sulfide
HPA: hypothalamus-pituitary-adrenal
IL-6: interleukin 6
LPS: lipopolysaccharide
Mn-SOD: manganese superoxide dismutase
NAD: nicotinamide adenine dinucleotide
NADP: NAD phosphate
NO: nitric oxide
NLRP3: NOD-like receptor family, pyrin domain containing 3
NF-κB: nuclear factor kappa B
NRF1: nuclear respiratory factor 1
NRF2: nuclear respiratory factor 2
OXPHOS: oxidative phosphorylation
GSSG: oxidized glutathione
PGC-1α: peroxisome proliferator-activated receptor gamma coactivator 1-alpha
PPARγ: peroxisome proliferator-activated receptor gamma
ROS: reactive oxygen species
RONS: reactive oxygen nitrogen species
GSH: reduced glutathione
SCFA: short chain fatty acid
SIRT1: silent regulator 1
SPF: specific pathogen-free
STAT3: signal transducer and activator of transcription 3
SREBP-1c: steroid response element binding protein-1c
SOD: superoxide dismutase
TLR4: toll-like receptor 4
TCA: tricarboxylic acid
T3: tri-iodothyronine (receptor 43)
TNFα: tumor necrosis factor alpha
UCP2: uncoupling protein 2.
